# Supplementary material for: Comparative Metabolomics of Early Development of the Parasitic Plants Phelipanche aegyptiaca and Triphysaria versicolor
Source: Metabolites. 2019 Jun 13;9(6):114. doi: 10.3390/metabo9060114 (PMC6630630; doi:10.3390/metabo9060114)
Supplement: Supplementary file 1 [file metabolites-09-00114-s001.zip › Supplemental Figure 1.pdf]

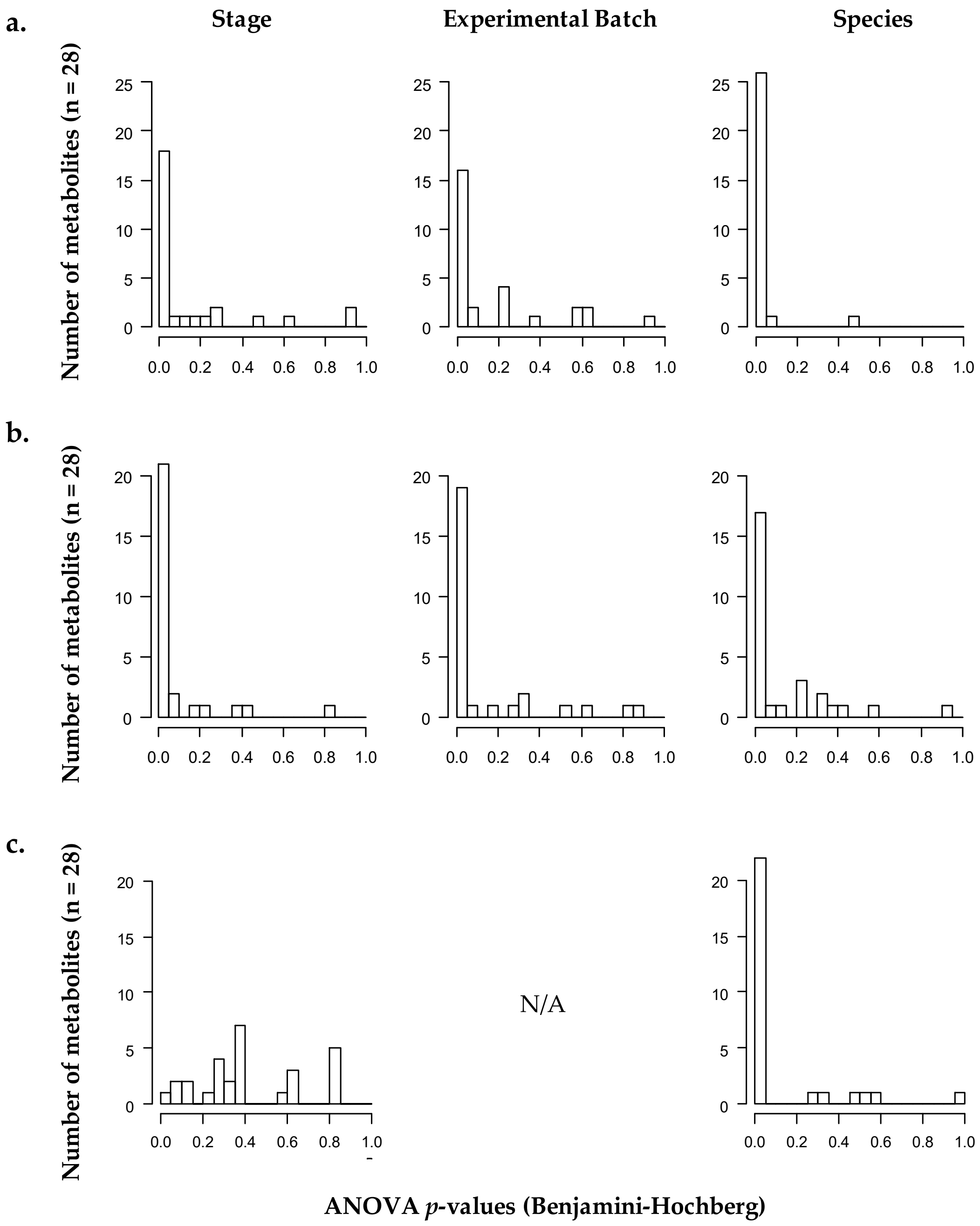

Figure S1. Histograms of *p*-values from ANOVA of metabolite levels. ANOVA was performed to evaluate the effect of stage, experimental batch, and species on each of 28 metabolites. Histograms of the Benjamini-Hochberg adjusted *p*-values are shown. (a) ANOVA performed with all data from three experimental batches. (b) ANOVA performed with data from the two experimental batches analyzing the *P. aegyptiaca – A. thaliana* system. (c) ANOVA performed with data from the *T. versicolor – M. truncatula* system. Because only one experiment was performed for the *T. versicolor – M. truncatula* system, experimental batch is not used as a factor in this ANOVA. For each table, half of the lowest measurement amount was added uniformly to each measurement in the raw data (0.25), and log10 transformed. ANOVA was performed using the MetabolomicsBasics package within R.
