# Supplementary material for: Comparative Metabolomics of Early Development of the Parasitic Plants Phelipanche aegyptiaca and Triphysaria versicolor
Source: Metabolites. 2019 Jun 13;9(6):114. doi: 10.3390/metabo9060114 (PMC6630630; doi:10.3390/metabo9060114)
Supplement: Supplementary file 1 [file metabolites-09-00114-s001.zip › Supplemental Figure 2.pdf]

P. aegyptiaca Experiment 1

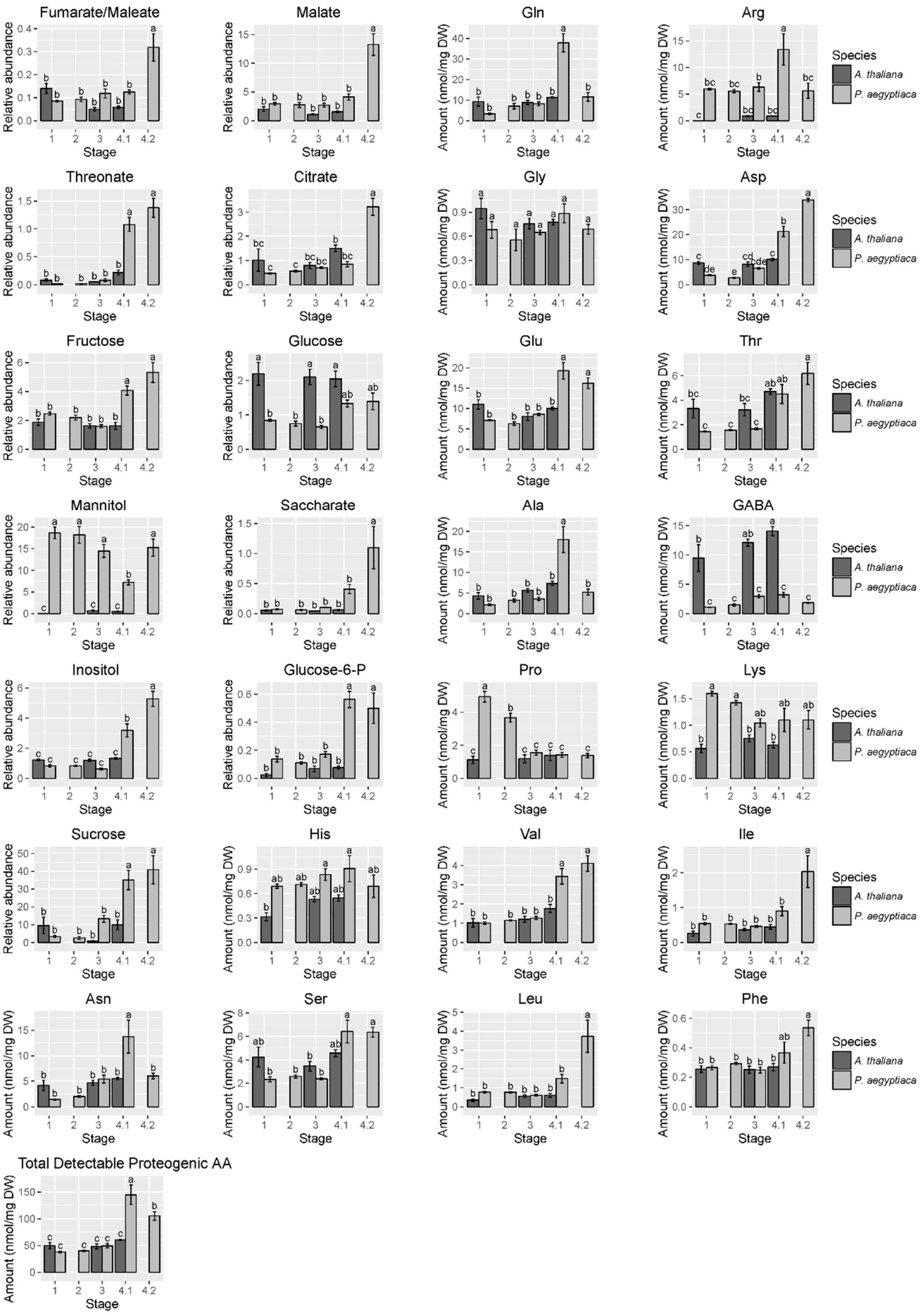

P. aegyptiaca Experiment 2

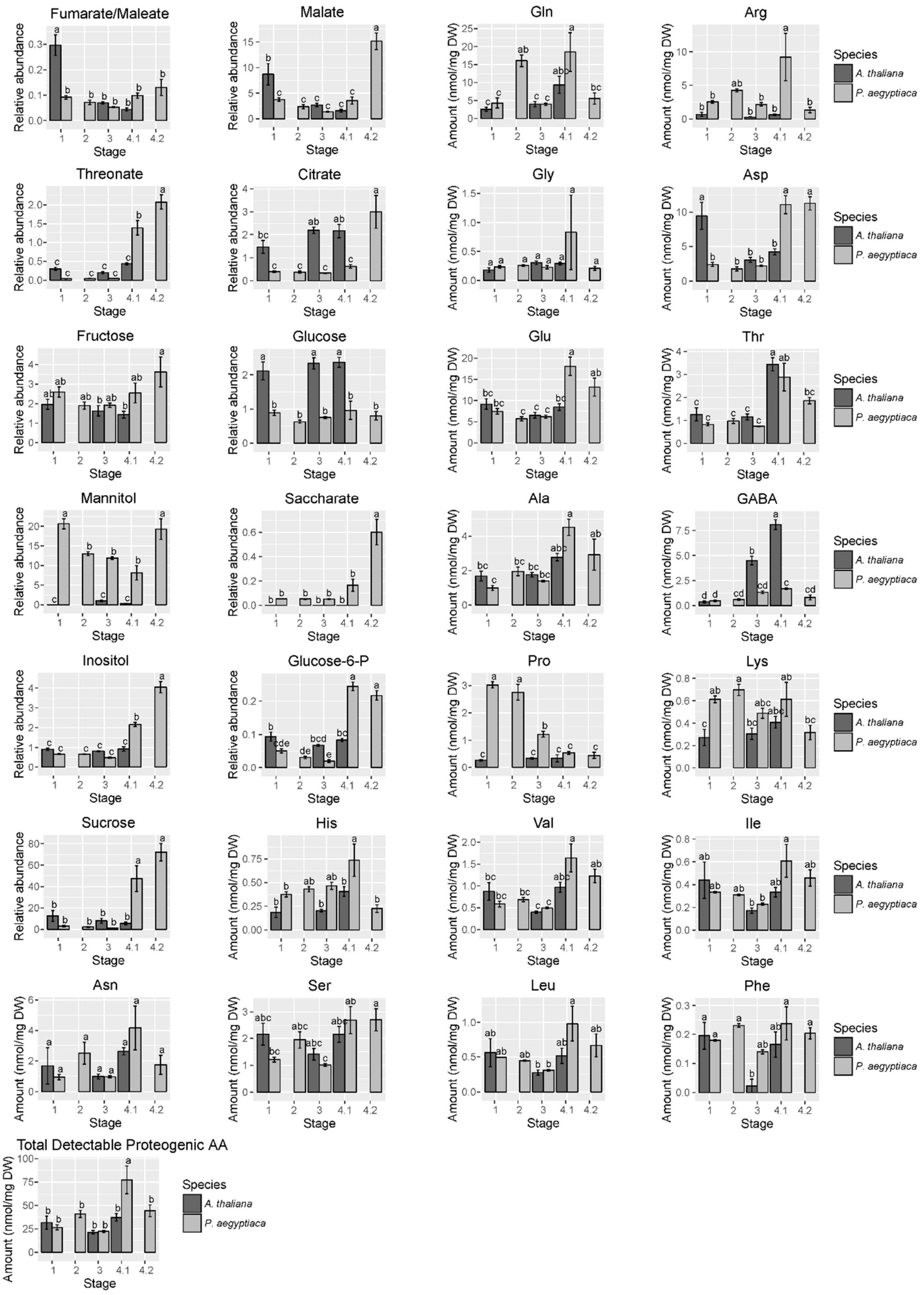

T. versicolor

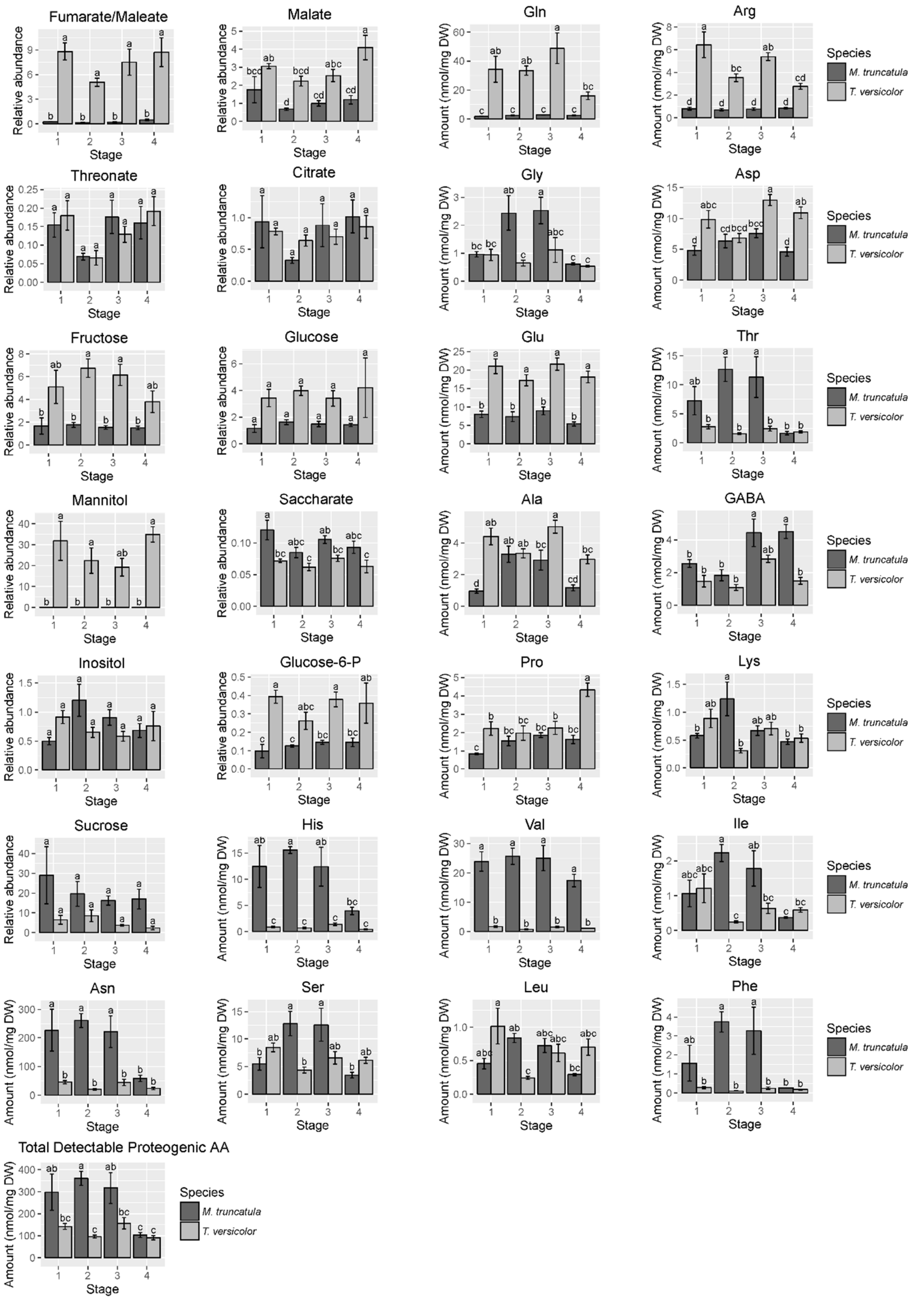

Figure S2. Abundance of quantified metabolites and total detectable protein coding amino acids. Data on *P. aegyptiaca* from the first and second experiment were graphed separately. Four to five biological replicates are used for each sample type. Amino acid abundance is expressed in nmol/mg DW. Sugar, carboxylic acid, and sugar alcohol abundance is expressed in relative abundance. In cases where *A. thaliana* host root was not collected for corresponding *P. aegyptiaca* stages, "NA" is shown. Tukey HSD connecting letters are given, run on host and parasite sample values concurrently. Standard error bars shown.
