# Supplementary material for: Comparative Metabolomics of Early Development of the Parasitic Plants Phelipanche aegyptiaca and Triphysaria versicolor
Source: Metabolites. 2019 Jun 13;9(6):114. doi: 10.3390/metabo9060114 (PMC6630630; doi:10.3390/metabo9060114)
Supplement: Supplementary file 1 [file metabolites-09-00114-s001.zip › Supplemental Figure 3.pdf]

A.

Asparagine synthase
Orthogroup 721

Class II

- AT5G65010.2
- AT5G10240.1
- PhAeBC5_6915.1
- TrVeBC3_4470.1

Class I

- AT3G47340.1
- PhAeBC5_258.1
- TrVeBC3_621.3

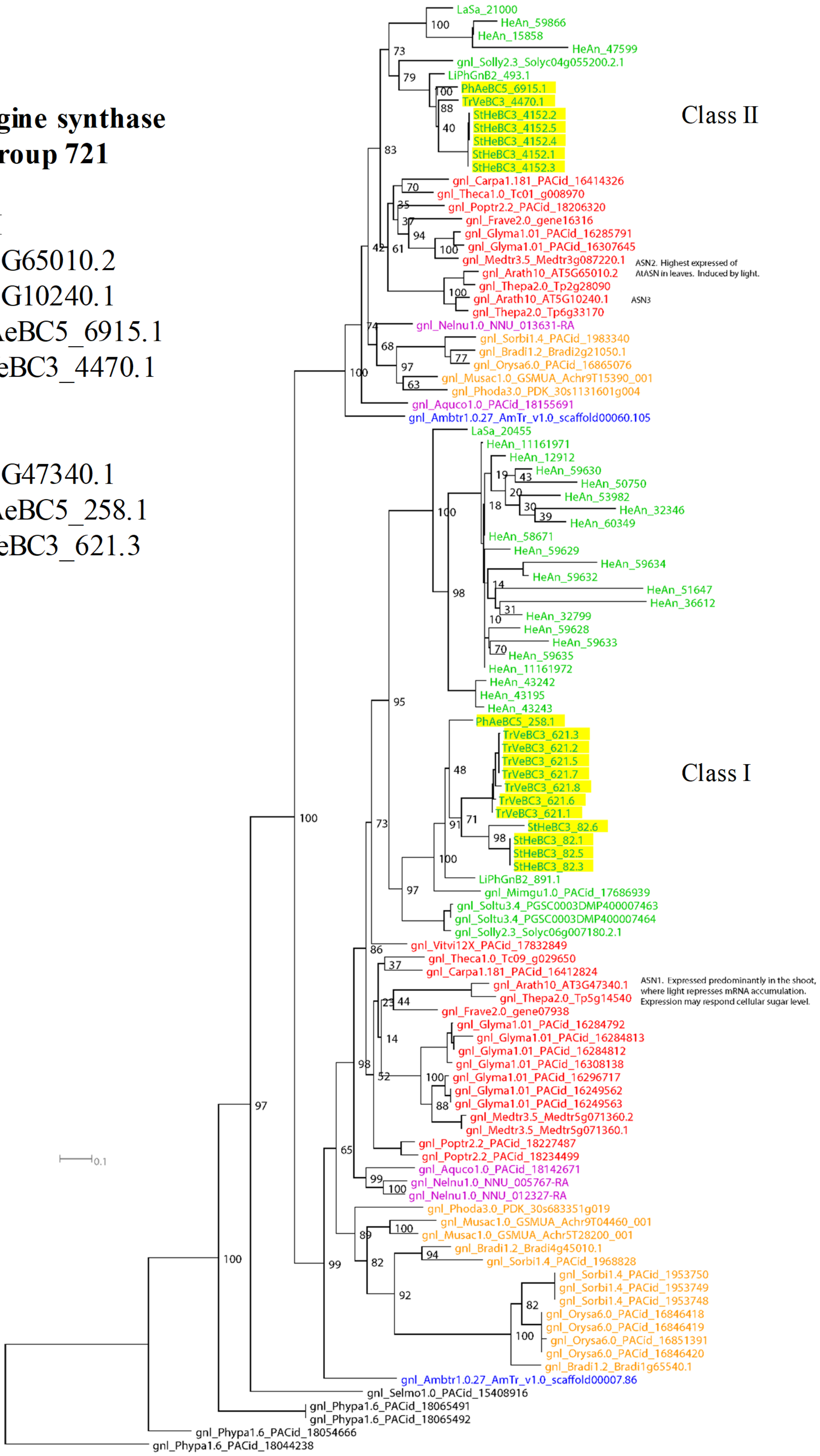

B.

Aspartate aminotransferase Orthogroup 2682

- AT2G22250.2
- OrAeBC5_520.1
- TrVeBC3_2708.1

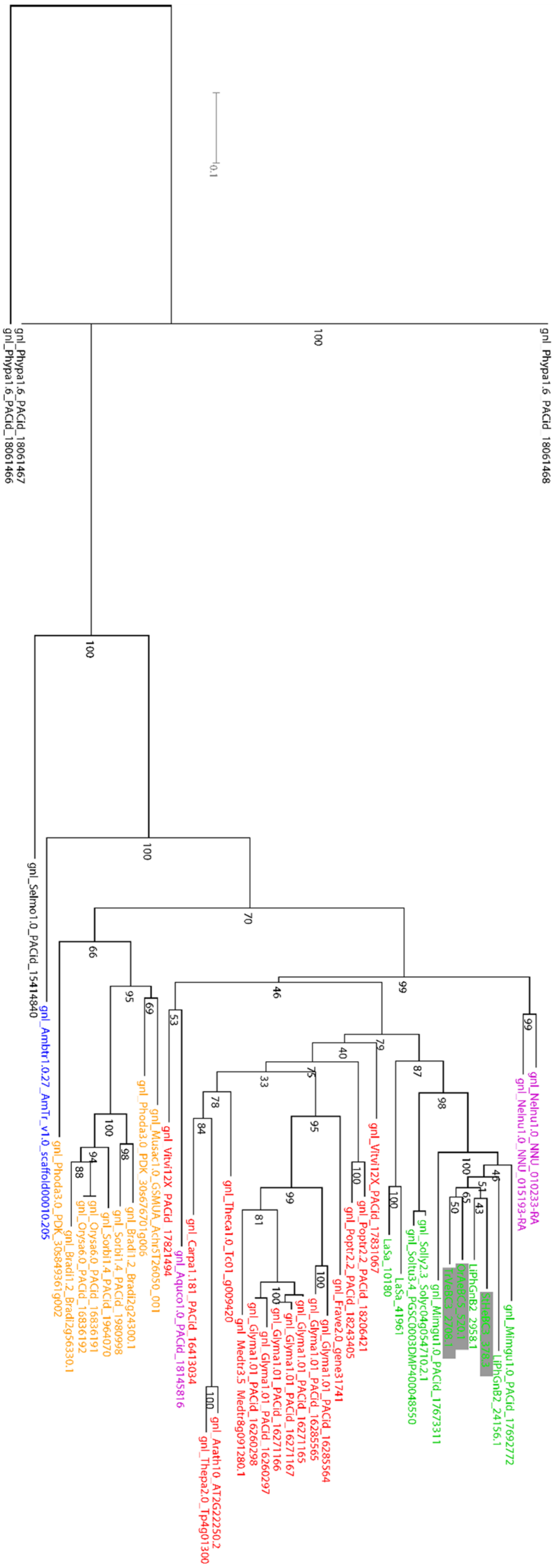

C.

Aspartate aminotransferase 1
Orthogroup 4541

- AT2G30970.1
- TrVeBC3_2450.7

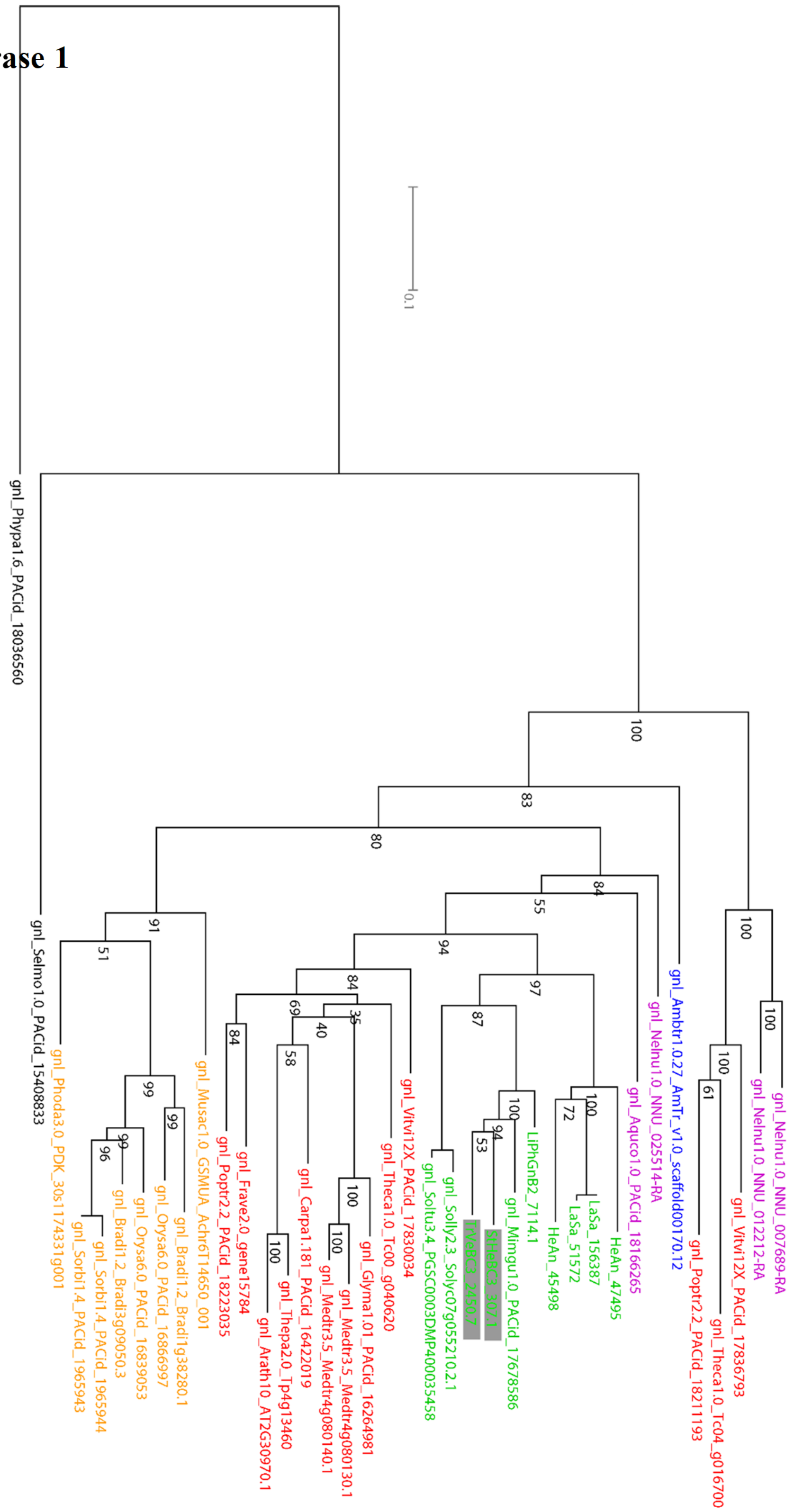

D.

Aspartate aminotransferase 2-4
Orthogroup 2325

- AT5G19550.1 (AAT2)
- AT1G62800.2 (AAT4)
- AT5G11520.1 (AAT3)
- OrAeBC5_403.1
- TrVeBC3_1788.2

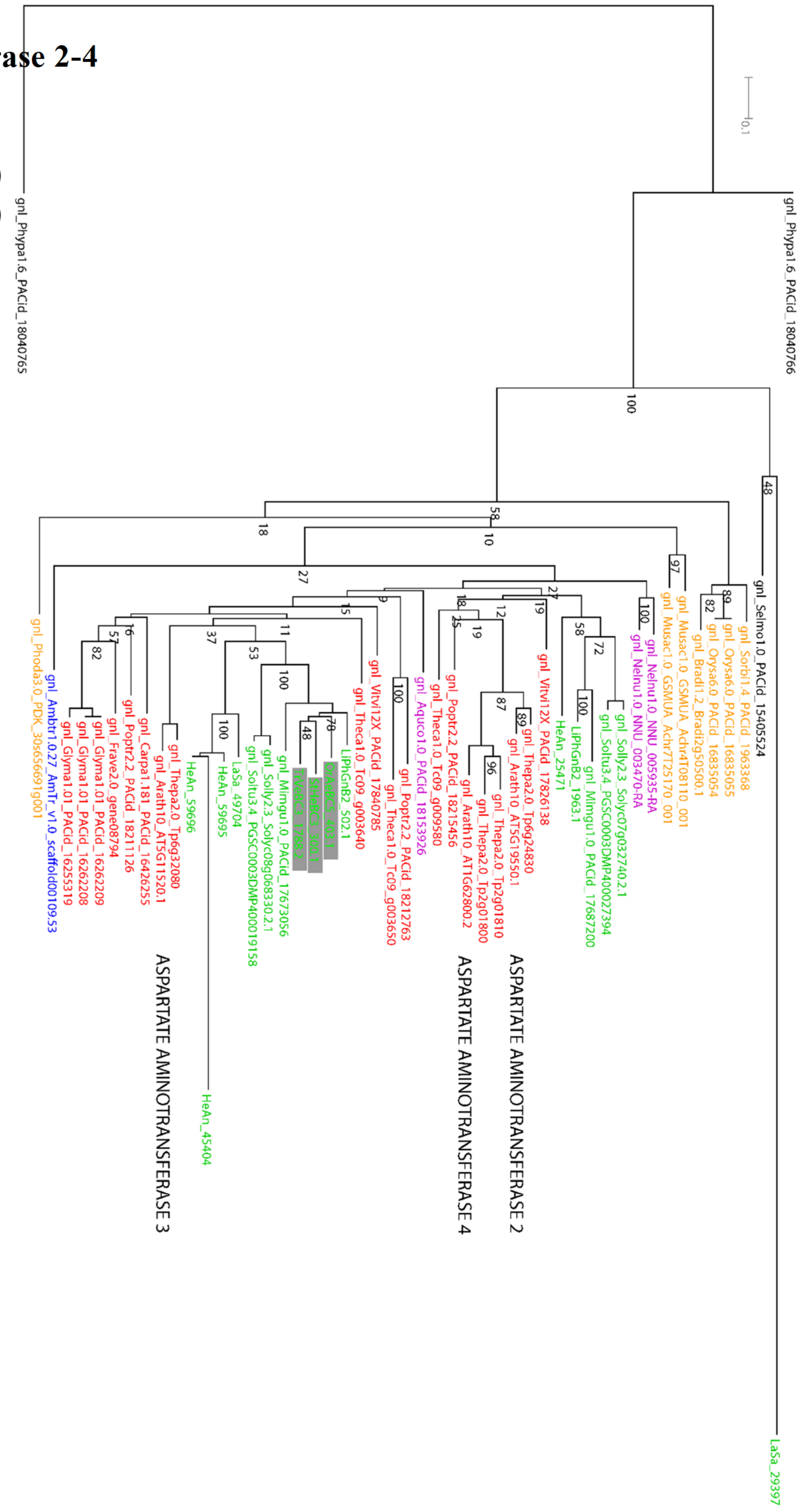

E.

Aspartate aminotransferase 5
Orthogroup 5243

- AT4G31990.3
- OrAeBC5_6581.1
- TrVeBC3_3479.1

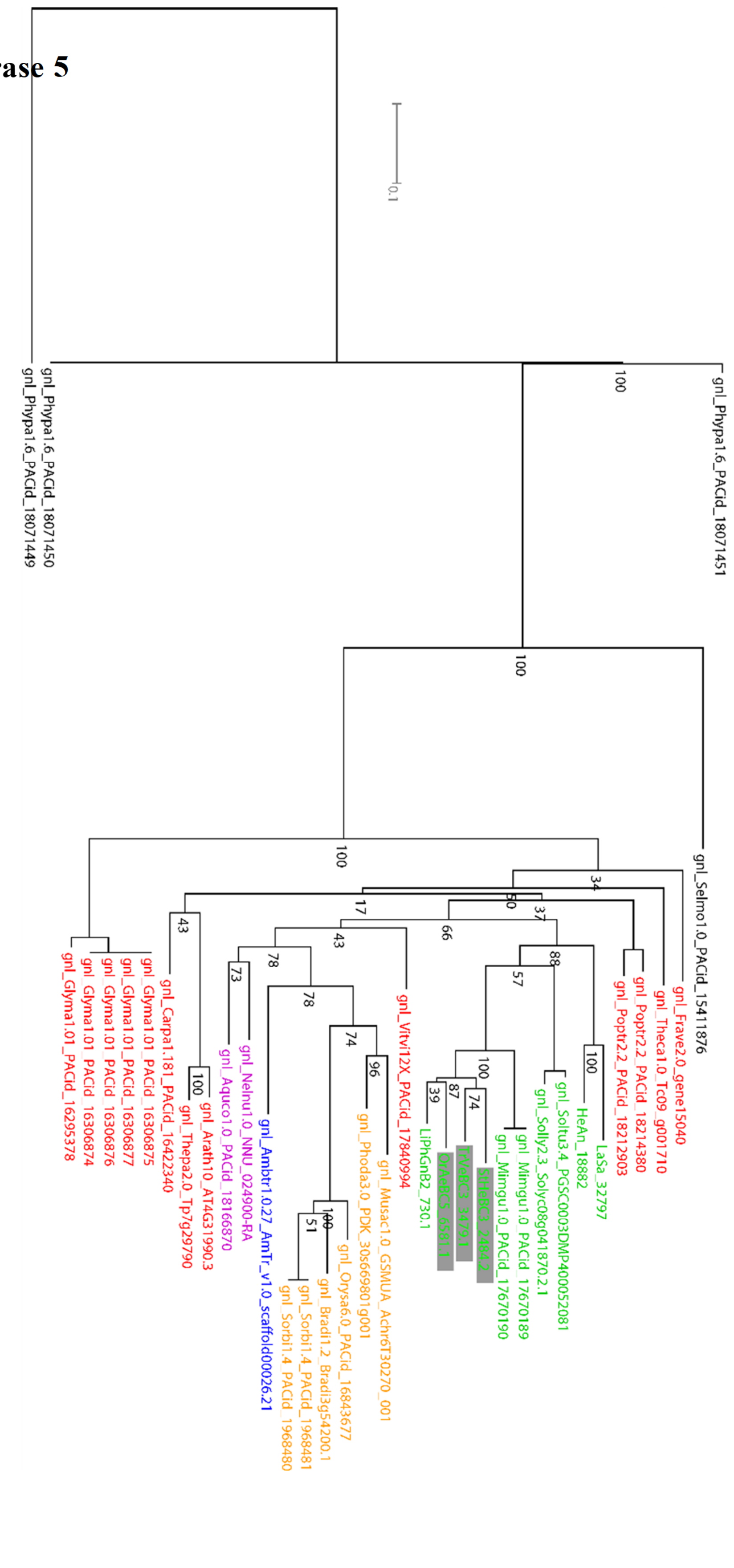

Figure S3. Phylogenetic trees of asparagine synthase and aspartate amino transferase homologs. Orthogroups and genes included in trees were determined using the PlantTribes database [39]. Orthogroups represent a PlantTribes delineated set of homologs and paralogs. Orobanchaceae homologs are highlighted in gray, and Orobanchaceae gene names and sequences are used as listed in the Parasitic Plant Genome Project database.
