# Supplementary material for: Comparative Metabolomics of Early Development of the Parasitic Plants Phelipanche aegyptiaca and Triphysaria versicolor
Source: Metabolites. 2019 Jun 13;9(6):114. doi: 10.3390/metabo9060114 (PMC6630630; doi:10.3390/metabo9060114)
Supplement: Supplementary file 1 [file metabolites-09-00114-s001.zip › Supplemental Table 2.pdf]

Species	Orthogroup	Orthogroup description	Contigs used from Yang 2015 paper
P. aegyptiaca	2325	Aspartate aminotransferase 3 - Chloroplastic (orthogroup also contains cytoplastic homologs in *A. thaliana*)	OrAeBC5_403.1
P. aegyptiaca	5243	Aspartate aminotransferase 5 - Chloroplastic	OrAeBC5_6581.1 - OrAeBC5_6581.9
P. aegyptiaca	4541	Aspartate aminotransferase 1 - Mitochondrial	OrAeBC5_36203.1, OrAeBC5_41726.1, OrAeBC5_49270.1, and OrAeBC5_53771.1
P. aegyptiaca	2682	Aspartate aminotransferase - Chlorplastic; prokaryotic-type	OrAeBC5_520.1
T. versicolor	2325	Aspartate aminotransferase 3 - Chloroplastic (orthogroup also contains cytoplastic homologs in *A. thaliana*)	TrVeBC3_1788.1 and TrVeBC3_1788.2
T. versicolor	5243	Aspartate aminotransferase 5 - Chloroplastic	TrVeBC3_3479.1
T. versicolor	4541	Aspartate aminotransferase 1 - Mitochondrial	TrVeBC3_2450.4, TrVeBC3_2450.5, TrVeBC3_2450.7, TrVeBC3_2450.8, TrVeBC3_2450.9, TrVeBC3_2450.10, TrVeBC3_2450.13, TrVeBC3_2450.14, and TrVeBC3_2450.15
T. versicolor	2682	Aspartate aminotransferase - Chlorplastic; prokaryotic-type	TrVeBC3_2708.1
P. aegyptiaca	721	Asparagine synthase - Class I	OrAeBC5_258.1
P. aegyptiaca	721	Asparagine synthase - Class II	OrAeBC5_6915.1
T. versicolor	721	Asparagine synthase - Class I	TrVeBC3_621.1 - TrVeBC3_621.8
T. versicolor	721	Asparagine synthase - Class II	TrVeBC3_4470.1

Table S2. Contigs used for expression analysis of two amino acid metabolism genes. Contigs and orthogroups described in Yang et al. 2015 [20] and used for expression analysis in Figure 8. Phylogenies of represented orthogroups are shown in Figure S2. Orthogroup descriptions are based on annotation of *A. thaliana* homologs present within the orthogroup. When multiple contigs are used, those contigs can be manually assembled and the sum of the FPKMs is used for analysis.
